# Self-growth of nitrogen-doped carbon nanotube composites using MOFs as templates and their application in Li-O_2_ batteries

**DOI:** 10.1039/d6ra06535j

**Published:** 2026-08-26

**Authors:** Yajun Zhao, Cen Wang, Zhipeng Yao, Guocai Zheng, Guofei Li, Xiaojing Li

**Affiliations:** a School of Automotive Applications and Rail Transit, Hefei Technology College Hefei 230012 Anhui China zyj@htc.edu.cn

## Abstract

The excessively high polarization potential during the charge and discharge process of Li-O_2_ batteries is one of the important factors restricting its development. Selecting a catalyst with bifunctional catalytic activity to improve the ORR and OER reaction kinetics is an important measure to reduce polarization and improve rate performance. In this study, Co-BTC was employed as a template to construct Ni-containing complexes *via* a hydrothermal process. During subsequent pyrolysis with dicyandiamide as the nitrogen source, nitrogen-doped carbon nanotubes were self-catalytically grown. The resulting structure effectively suppresses the charge–discharge polarization of Li-O_2_ batteries. When used as a cathode catalyst, Ni/Co_2_N@NCNTs batteries can stably cycle 220 times, Ni@NCNTs can only cycle 70 times, and Co_2_N@NCNTs can only cycle 110 times.

## Abstract 2

Engineered Ni/Co_2_N@NCNTs unlocks synergistic dual-site catalysis for high-performance Li-O_2_ batteries.

## Graphical abstract

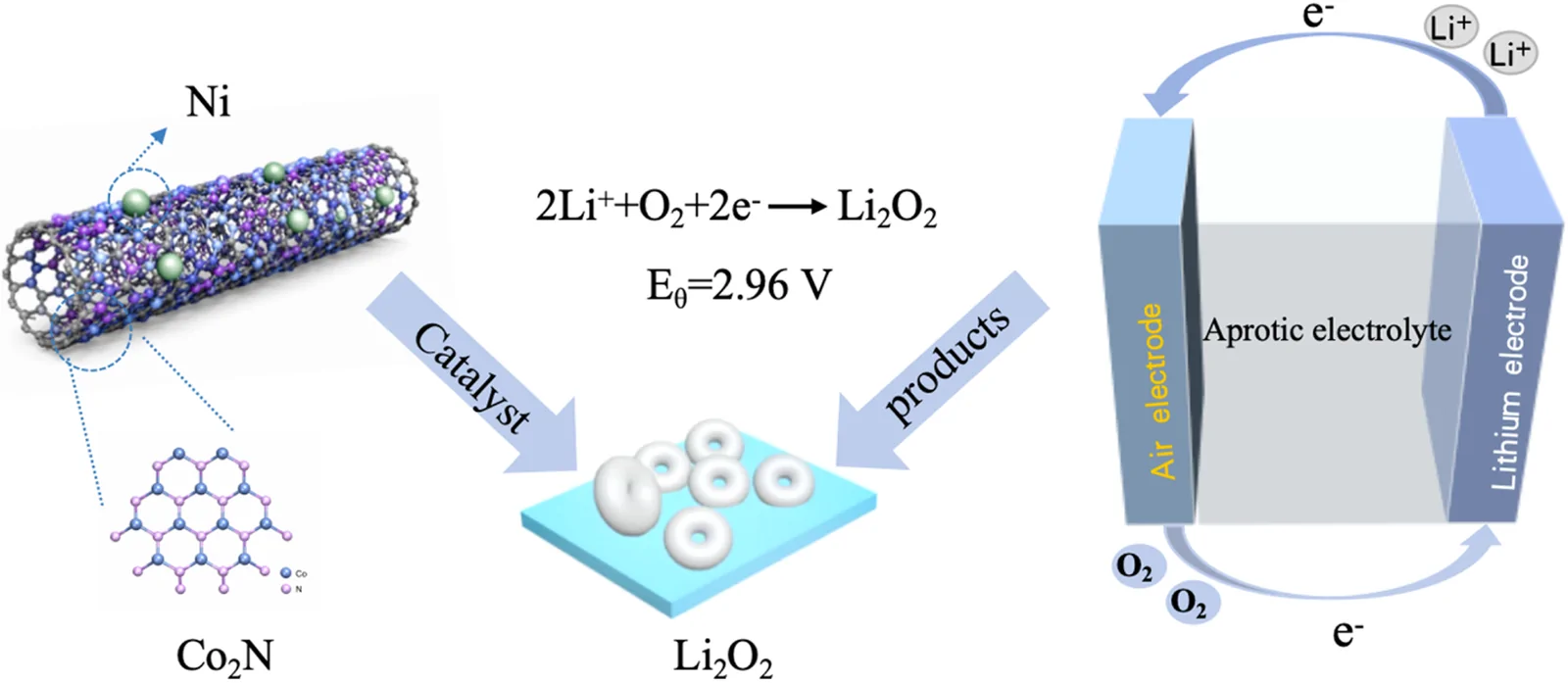

## Introduction

As industrial production continues to progress, the consumption of fossil fuels is escalating, exacerbating the severity of environmental pollution. Consequently, developing highly efficient and sustainable energy technologies has become an urgent research priority.^1–3^ Li-O_2_ batteries represent a novel chemical energy storage technology that operates on the lithium-oxygen reaction occurring at the electrode surface. With a theoretical specific energy density of 3500 Wh kg^−1^, they have the potential to effectively meet the energy storage demands of industrial production.^4–6^ However, the slow kinetics of the oxygen reduction reaction (ORR) and oxygen evolution reaction (OER) in lithium-oxygen batteries and the low decomposition efficiency of the discharge product Li_2_O_2_ ultimately limit the practical application of lithium-oxygen batteries.^7–9^ In recent years, extensive studies have explored various catalysts, including noble metals and their oxides, carbon-based materials, transition metal compounds, metal nitrides, and metal carbides, to enhance the reaction kinetics of lithium-oxygen batteries.^10–12^

Among various nitrogen-containing carbon materials, nitrogen-doped graphene has been extensively investigated as a bifunctional electrocatalyst for ORR and OER owing to its unique electronic structure and structural advantages.^13^ However, due to the strong van der Waals interactions between adjacent layers, graphene assemblies are prone to aggregation or restacking, which ultimately deteriorates the electrochemical activity of the catalyst.^14^ Nitrogen-doped carbon nanotubes (CNTs) wrapped with metal alloys have emerged as a promising alternative to traditional CNTs in recent years. This combination not only enhances electron transfer but also generates additional active sites for electrochemical reactions.^15^ Therefore, it is highly attractive to fabricate nitrogen-doped carbon nanotubes (N-doped CNTs) wrapped with metal alloys with optimized porosity and abundant active sites through a simple and cost-effective strategy to improve the catalytic performance of these materials.^16^

Metal organic frameworks (MOFs) can provide a large number of active sites, which are conducive to the adsorption and diffusion of reactant molecules and improve catalytic efficiency. At the same time, selective catalysis of specific molecules can be achieved by regulating the size and shape of the pores.^17–19^ Studies have shown that the three-dimensional porous carbon network structure obtained by pyrolysis of MOF can significantly improve the electrochemical energy storage capacity of the battery. The large specific surface area of these materials enables more abundant diffusion pathways and exceptional conductivity, features that are challenging to achieve with other types of carbon-based structures.^20,21^ Transition metals possess the ability to self-catalyze the formation of carbon nanotubes. Utilizing cobalt-based metal–organic frameworks (MOFs) as templates and leveraging these self-catalytic properties to prepare nitrogen-doped carbon nanotube materials with encapsulated metals is an advantageous strategy for enhancing catalytic activity.

Recent studies in oxygen electrocatalysis have revealed that electronic structure modulation and interface engineering are effective approaches for improving ORR/OER kinetics by regulating electron transfer and oxygen intermediate adsorption.^22,23^ Inspired by these advances, constructing transition-metal-based heterostructures with strong interfacial coupling provides a promising strategy for Li-O_2_ batteries. The integration of conductive metallic components and catalytically active nitrides is expected to synergistically enhance oxygen redox kinetics and promote reversible Li_2_O_2_ formation/decomposition.

In this chapter, Co^2+^ and 1,3,5-benzenetricarboxylic acid were employed as metal ligands to synthesize the Co-BTC metal–organic framework (MOF) template. Nitrogen-doped carbon nanotubes were self-catalytically generated using the Co-MOF/Ni(OH)_2_ template through a combination of hydrothermal synthesis and high-temperature pyrolysis. These materials were utilized as cathode catalysts for Li-O_2_ batteries, which exhibited an exceptionally high discharge specific capacity and significantly enhanced cycling performance. This demonstrates their outstanding bifunctional catalytic activity for both the oxygen reduction reaction (ORR) and oxygen evolution reaction (OER).

## Experimental section

### Preparation of Ni/Co_2_N@NCNTs

A certain amount of Co-BTC was dissolved in 40 mL of deionized water and 40 mL of ethanol. 0.485 g of NiCl_2_·6H_2_O, 0.2315 g of ammonium fluoride, and 0.546 g of urea were added under stirring. The mixture was stirred for 30 min and transferred to a 100 mL Teflon reactor. The mixture was hydrothermally reacted at 180 °C for 12 h. After cooling to room temperature, the mixture was centrifuged, washed, and the precipitate was collected and dried. A certain amount of dicyandiamide was weighed according to the mass ratio of product to dicyandiamide of 1 : 20. The two were placed in two quartz boats respectively. The dicyandiamide was placed in the upwind side of the tube furnace and the product was placed in the downwind side. The mixture was calcined in an Ar atmosphere. The temperature was first increased to 400 °C at 10 °C min^−1^ and kept at this temperature for 2 h. Then, the temperature was increased to 800 °C at 10 °C min^−1^ and kept at this temperature for 4 h. The Ni/Co_2_N@NCNTs composite material was obtained.

## Results and discussion

**Fig. 1:**
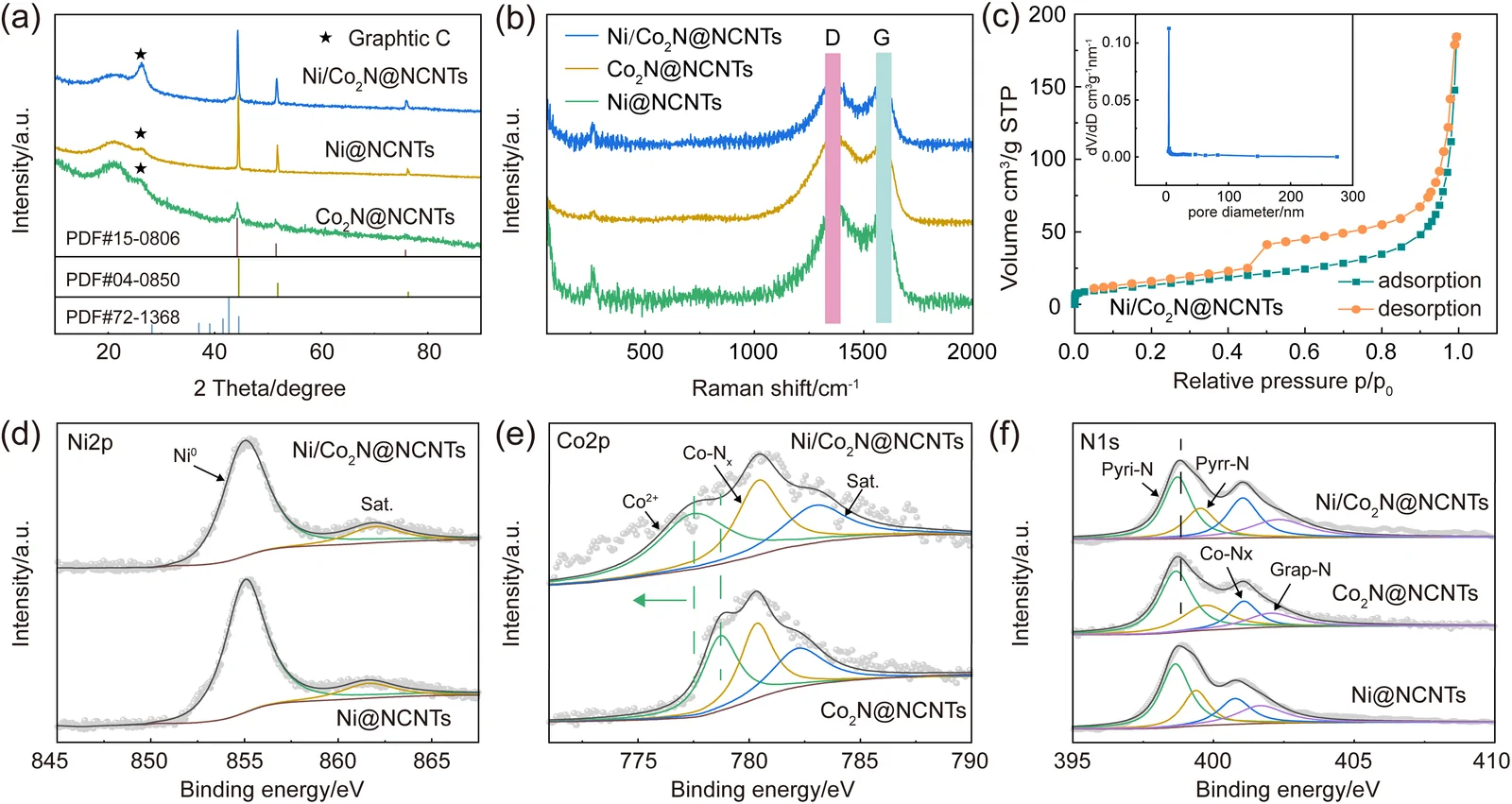
(a) XRD patterns of three materials; (b) Raman curves of three materials; (c) N_2_ adsorption–desorption curves of Ni/Co_2_N@NCNTs; (d) The XPS spectrum of Ni 2p of Ni@NCNTs and Ni/Co_2_N@NCNTs; (e) the XPS spectrum of Co 2p of Co_2_N@NCNTs and Ni/Co_2_N@NCNTs; (f) the XPS spectrum of N 1s of three materials.

X-ray diffraction (XRD) measurements were conducted to identify the crystalline structure and phase composition of the material. In Fig. 1a, both Co_2_N@NCNTs and Ni/Co_2_N@NCNTs exhibit a distinct diffraction peak at 2*θ* = 44.5°, corresponding to the (021) plane of Co_2_N (JCPDS No. 72-1368). In addition, the peak at 26.3° corresponds to graphitic carbon (JCPDS No. 01-0640), which is associated with the (002) crystal plane. The peaks observed for Co_2_N@NCNTs at 51.5° and 75.9° correspond to metallic cobalt (JCPDS No. 15-0806), while the peaks observed for Ni@NCNTs at 51.6° and 76.1° correspond to metallic nickel (JCPDS No. 04-0850). The diffraction peaks of Ni/Co_2_N@NCNTs align with those of both components, confirming the successful synthesis of the Ni/Co_2_N@NCNTs material.

The degree of graphitization of the material was analyzed by Raman spectroscopy. For all samples, the D band, located at approximately 1339 cm^−1^, corresponds to structural defects in the graphite framework, while the G band, situated at around 1590 cm^−1^, represents the first-order scattering of the E_2g_ mode observed in the sp^2^ carbon domain (Fig. 1b).^24^ After calculation, the strength ratio (*I*_D_/*I*_G_) of Ni@NCNTs and Co_2_N@NCNTs materials is about 1.01, and the strength ratio (*I*_D_/*I*_G_) of Ni/Co_2_N@NCNTs material is about 0.96, suggests the presence of a short-range ordered graphite structure in these three materials.^25^ Compared to Ni@NCNTs and Co_2_N@NCNTs materials, the *I*_D_/*I*_G_ value of the Ni/Co_2_N@NCNTs material decreases, indicating an enhanced degree of graphitization after Ni doping.^26^ This observation is in good agreement with the XRD results.

The porous structures of Ni@NCNTs, Co_2_N@NCNTs, and Ni/Co_2_N@NCNTs were further investigated through N_2_ adsorption/desorption curves. At a relative pressure of *P*/*P*_0_ < 0.05, typical type IV adsorption–desorption isotherms with distinct hysteresis loops are observed for all three materials (Fig. 1c and S1), indicating the presence of hierarchical porous structures in the BTC-derived samples. Notably, the pore size distribution indicates that Ni/Co_2_N@NCNTs have a larger pore size (3.831 nm) compared to Ni@NCNTs (3.808 nm) and Co_2_N@NCNTs (3.824 nm). The plentiful hierarchical pores in NCNTs not only improve electrolyte penetration and facilitate the transport of Li^+^ and electrons but also offer ample space for the conversion processes of O_2_ and Li_2_O_2_. This structural advantage is beneficial for improving the electrochemical performance of LOBs.^27,28^ Using the BET model to calculate the specific surface areas of the three materials, it was found that Ni@NCNTs has a specific surface area of 98.6 m^2^ g^−1^, Co_2_N@NCNTs has 83.5 m^2^ g^−1^, and Ni/Co_2_N@NCNTs has 49.8 m^2^ g^−1^. Compared to the other two materials, the specific surface area of Ni/Co_2_N@NCNTs is slightly reduced. This reduction may be attributed to pore blockage on the surface during the autocatalytic process or the collapse of micropores during the secondary calcination process.

Further insight into the surface chemical composition and bonding configurations of the prepared samples was obtained through X-ray photoelectron spectroscopy (XPS) analysis. The Ni 2p spectra of both Ni@NCNTs and Ni/Co_2_N@NCNTs reveal a peak at 856.5 eV, corresponding to Ni 2p_3/2_, and a derived satellite peak at 862.3 eV. There are no diffraction peaks indicating Ni in other valence states, confirming that Ni exists as an elemental substance in these two materials (Fig. 1d).^29^ As shown in Fig. 1e, the high-resolution Co 2p spectra of Co_2_N@NCNTs and Ni/Co_2_N@NCNTs reveal that the peak at 778.6 eV is attributed to Co^2+^, while the peak at 781.2 eV corresponds to Co–N_*x*_ interactions. Additionally, the peak at 783.2 eV is identified as a derived satellite peak.^30–32^ The Co 2p peaks exhibit a negative binding energy shift relative to Co_2_N@NCNTs, indicating an increased electron density around Co species. This shift can be attributed to interfacial electronic interaction between metallic Ni and Co_2_N, leading to charge redistribution and modulation of the Co electronic environment. Such electronic coupling is beneficial for optimizing oxygen intermediate adsorption and accelerating reversible oxygen redox reactions. The XPS spectra of N 1s for the three materials (Fig. 1f) can be categorized into three distinct states of nitrogen. The peak at 397.9 eV corresponds to pyridinic nitrogen, while the peak at 398.6 eV is attributed to the Co–N_*x*_ bond. Additionally, the peak at 399.8 eV is associated with pyrrolic nitrogen, and the peak at 400.8 eV corresponds to graphitic nitrogen.^33^ By analyzing the peak areas to determine their relative proportions, it was calculated that the pyridine N content in Ni@NCNTs was 41.7%, the pyrrole N content was 18.3%, and the graphite N content was 18.9%. The pyridine N content in Co_2_N@NCNTs was 16.3%, and the pyrrole N content was 43.6%, the graphite N content was 14.9%, the pyridine N content in Ni/Co_2_N@NCNTs is 35.3%, the pyrrole N content is 25.2%, and the graphite N content is 20.7%. Previous studies have indicated that graphitic nitrogen plays a crucial role in improving the ORR performance by aiding electron transfer throughout the process. Consequently, a higher concentration of graphitic nitrogen can boost ORR activity.^34,35^

**Fig. 2:**
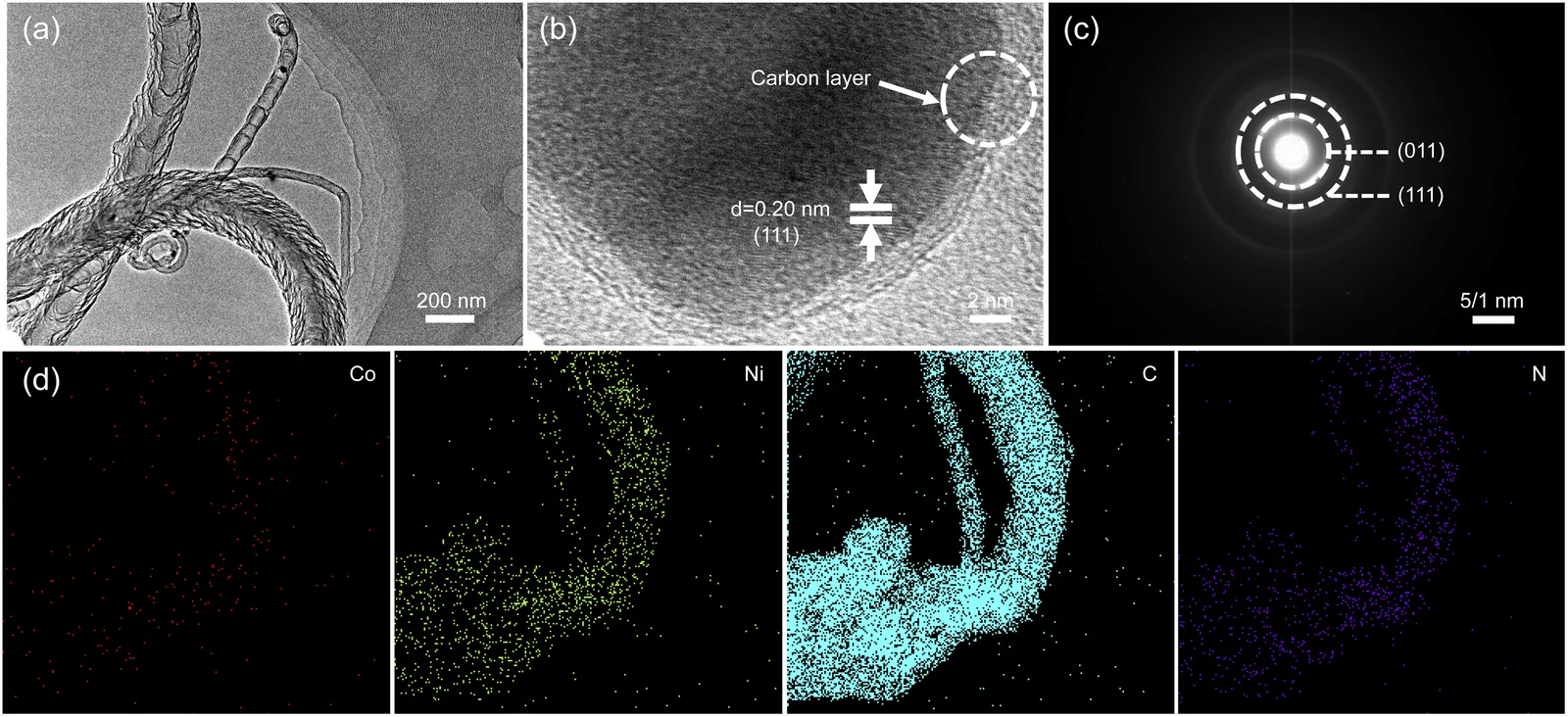
Images of Ni/Co_2_N@NCNTs: (a) TEM; (b) HRTEM; (c) SAED; (d) EDX mapping.

The appearance and morphology of the materials were observed by SEM and TEM. As shown in Fig. S2a, Ni@NCNTs has a carbon nanotube structure, and using Co-BTC as a template, the Co_2_N@NCNTs material and Ni/Co_2_N@NCNTs material after nitriding with dicyandiamide are also relatively uniform carbon nanotube structure (Fig. S2b and c). The TEM image further shows that the Ni/Co_2_N@NCNTs material is a self-grown carbon nanotube structure (Fig. 2a). HRTEM was used to measure the lattice fringe spacing and it was found that the lattice fringe spacing was 0.204 nm, corresponding to the (111) crystal plane of Ni (Fig. 2b). The lattice spacings obtained from SAED are 0.316 nm and 0.204 nm, corresponding to the (011) plane of Co_2_N and the (111) plane of Ni, respectively (Fig. 2c). The EDX mapping results show that there are four elements, Co, Ni, C, and N, in the material, and the elements are evenly distributed, suggesting the intimate coupling between Ni and Co2N within the carbon nanotube framework (Fig. 2d).

**Fig. 3:**
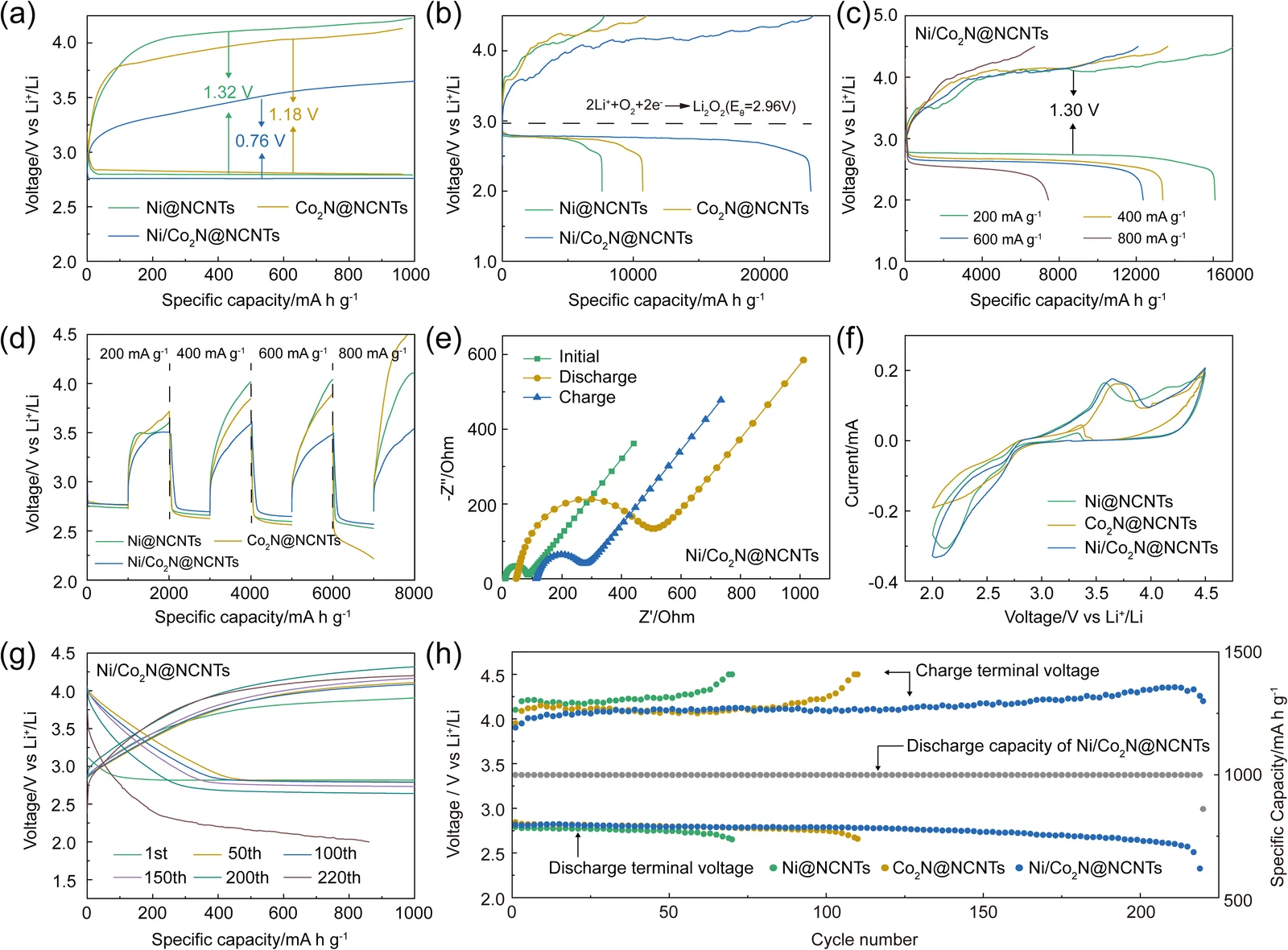
Battery performance diagrams of three materials: (a) overvoltage; (b) capacity; (c) rate performance diagram of Ni/Co_2_N@NCNTs; (d) comparison chart of the rate charge and discharge termination voltage of the three materials; (e) the impedance change diagram of Ni/Co_2_N@NCNTs in different states; (f) CV diagram of the three materials; (g) the battery cycle diagram of Ni/Co_2_N@NCNTs; (h) comparison diagram of the cycle termination voltage of the three materials.

The electrochemical properties of the materials were tested by assembling CR2032 button cells. As shown in Fig. 3a, the overvoltage of the battery using Ni@NCNTs, Co_2_N@NCNTs, and Ni/Co_2_N@NCNTs as cathode catalysts is 1.32 V, 1.18 V, and 0.76 V respectively, proving that Ni/Co_2_N@NCNTs is used as the cathode catalyst. LOBs catalyst can effectively reduce polarization during charge and discharge processes. Similarly, the battery capacity using this as a catalyst has also been greatly improved. When tested at 100 mA g^−1^, the Co_2_N@NCNTs electrode delivered a high discharge specific capacity of 10 721.6 mAh g^−1^, considerably exceeding the 7612.6 mAh g^−1^ obtained for the Ni@NCNTs counterpart, while the discharge specific capacity of Ni/Co_2_N@NCNTs material is 23 568.2 mAh g^−1^ (Fig. 3b).

The electrochemical charge–discharge behavior of the three catalysts was systematically investigated at current densities ranging from 200 to 800 mA g^−1^, as shown in Fig. 3c, d and S3. The discharge specific capacities for Ni@NCNTs are recorded as 7226.6, 5613.7, and 3672.5 mAh g^−1^, respectively (Fig. S3a). For Co_2_N@NCNTs, these values are 8484.8, 6567.1, and 5722.5 mAh g^−1^, respectively (Fig. S3b). In contrast, the Ni/Co_2_N@NCNTs materials show discharge specific capacities of 16 097.4, 13 378.7, 12 356.9 and 7439.7 mAh g^−1^ at the same current densities (Fig. 3c). This demonstrates that Ni/Co_2_N@NCNTs can contribute to a notable enhancement in the rate capability of LOBs when used as a catalyst. Comparing the overpotentials of the three materials at 200 mA g^−1^, it can be found that the Ni/Co_2_N@NCNTs material still has the smallest overpotential of 1.30 V, which is lower than the 1.34 V of Ni@NCNTs and the 1.45 V of Co_2_N@NCNTs, indicating that the polarization degree of Ni/Co_2_N@NCNTs material under high current is still relatively low. At the same time, the cut-off voltages of Ni@NCNTs, Co_2_N@NCNTs, and Ni/Co_2_N@NCNTs were compared under different current densities when charging and discharging to 1000 mAh g^−1^. It was found that the cut-off voltages of Ni/Co_2_N@NCNTs electrode was 2.77/3.51 V, 2.70/3.59 V, 2.65/3.49 V, 2.57/3.55 V, which was significantly better than the other two materials (Fig. 3d).

Electrochemical impedance spectroscopy (EIS) was employed to record and analyze the impedance changes of Li-O_2_ batteries based on Ni@NCNTs, Co_2_N@NCNTs, and Ni/Co_2_N@NCNTs at different discharge/charge stages (Fig. 3e and S4). In the equivalent circuit, *R*_o_ signifies the intrinsic resistance of the Li-O_2_ battery. The semicircle observed in the intermediate frequency range corresponds to *R*_ct_ (charge transfer resistance) and *C*_dl_ (double layer capacitance). The line appearing in the low-frequency range is indicative of W (Warburg impedance), resulting from the hindrance to oxygen diffusion to the electrode and lithium ion movement.^36,37^ Findings highlight that *R*_ct_ exhibits a more pronounced variation compared to *R*_o_ and *C*_dl_ across various battery phases. Typically, battery impedance rises post-discharge due to Li_2_O_2_ formation, and drops post-charge as Li_2_O_2_ decomposes. For Ni/Co_2_N@NCNTs, *R*_ct_ impedance escalates from 70 Ω to 383 Ω following discharge, then reverts to 143 Ω post-charge, nearly reaching its original value (Fig. 3e), suggesting near-complete breakdown of discharge byproducts. In contrast, Ni@NCNTs and Co_2_N@NCNTs show greater *R*_ct_ increases, with Ni@NCNTs rising from 114 Ω to 467 Ω (Fig. S4a) and Co_2_N@NCNTs from 72 Ω to 624 Ω. Post-charge, impedances stabilize at 204 and 233 Ω, respectively (Fig. S4b). The EIS data for the trio of catalysts reveal that the Li_2_O_2_ on the surface of Ni/Co_2_N@NCNTs is largely decomposed post-charge. Conversely, Li_2_O_2_ on Ni@NCNTs and Co_2_N@NCNTs surfaces is only partially decomposed, leading to elevated *R*_ct_ values. Given the significance of *R*_ct_ in charge transfer, this pattern suggests Ni/Co_2_N@NCNTs excels in facilitating charge transfer and enhancing the reversibility of Li-O_2_ batteries. In order to further analyze the ORR/OER catalytic performance of the three materials, the CV test was scanned at a rate of 0.1 mV s^−1^ in the range of 2–4.5 V. The results are shown in Fig. 3f. The results show that the Ni/Co_2_N@NCNTs cathode shows a lower OER (Li_2_O_2_ → O_2_ + 2Li^+^ + 2e^−^) potential and a higher ORR (O_2_ + 2Li^+^ + 2e^−^ → Li_2_O_2_) potential, indicating that the redox kinetics of Ni/Co_2_N@NCNTs is better.^38^ At the same time, for Ni/Co_2_N@NCNTs, a clear cathode peak corresponding to the formation of Li_2_O_2_ can be identified at 2.17 V, and the peak potential reached is higher than that of Ni@NCNTs and Co_2_N@NCNTs, indicating that the ORR kinetics have been improved. There is a clear anodic peak at 3.75 V, corresponding to the decomposition of Li_2_O_2_. Most notably, the ORR/OER peak current and integrated area of Ni/Co_2_N@NCNTs exhibit significant improvement compared to Ni@NCNTs and Co_2_N@NCNTs. This enhancement indicates that a greater amount of Li_2_O_2_ can be generated during discharge and that Li_2_O_2_ decomposes more readily during charge, demonstrating excellent oxidation/reduction reversibility.^39^

The cycle performance of the battery was shown in Fig. 3g, h and S5. Due to the large charge and discharge polarization, the reversibility of the battery is poor. LOBs with Ni@NCNTs as catalyst can only cycle 70 times (Fig. S5a), Co_2_N@NCNTs can cycle 110 times (Fig. S5b), and LOBs with Ni/Co_2_N@NCNTs as catalyst can stably cycle 220 times (Fig. 3g). Simultaneously, by analyzing the charge and discharge termination voltage during the cycle, it can be found that the battery with Ni@NCNTs and Co_2_N@NCNTs as catalysts has poor cycle stability and quickly reaches the upper and lower limits of charge and discharge in a short time. However, the battery with Ni/Co_2_N@NCNTs as catalyst has a stable voltage in the first 220 cycles without significant fluctuations (Fig. 3h), indicating that the battery has good cycle stability. It also further illustrates that the composite catalyst prepared by self-grown carbon nanotubes using Co-BTC as a template can enhance the ORR and OER reaction kinetics, accelerate the generation and decomposition of the discharge product Li_2_O_2_, and thus greatly improve the battery cycle performance.

**Fig. 4:**
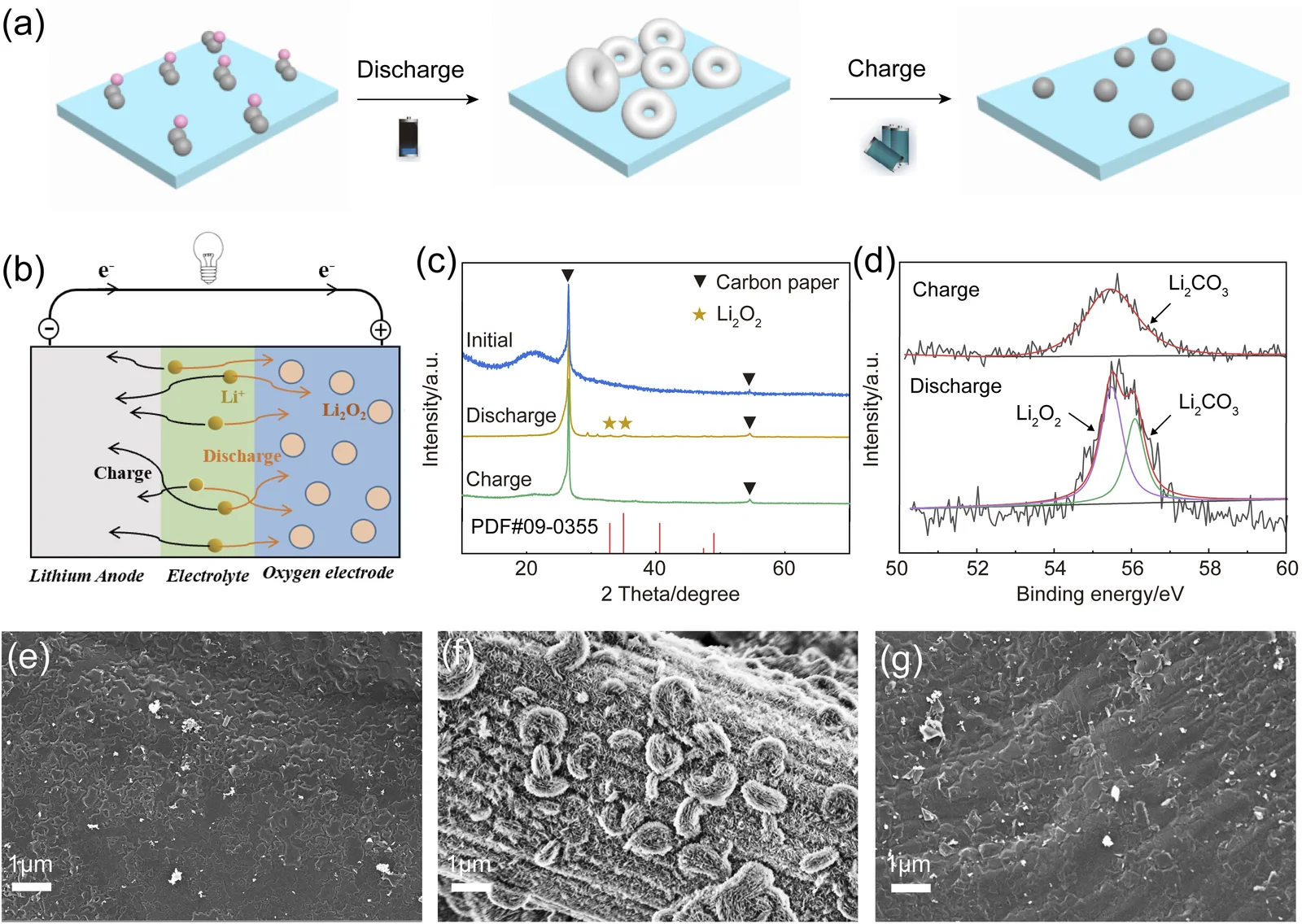
(a) Schematic diagram of the charging and discharging process of Li-O_2_ battery; (b) Reaction mechanism diagram of Li-O_2_ battery; (c) XRD curves of carbon sheet in different states; (d) XPS spectrum of Li 1s of carbon sheet in different states; SEM of carbon sheet in different states: (e) initial; (f) after discharging; (g) after charging.

Li-O_2_ batteries employ metallic lithium for the anode and oxygen for the cathode. Within a non-protonic electrolyte, the discharge process can yield either Li_2_O_2_ or Li_2_O. Nonetheless, Li_2_O formation is less desirable due to the substantial bond energy of the O–O bond in O_2_, which makes it difficult to cleave, and Li_2_O's inherently low electronic conductivity both internally and on its surface.^40^ Furthermore, Li_2_O_2_ offers greater reversibility compared to Li_2_O in the non-aqueous Li-O_2_ battery setting. Therefore, Li_2_O_2_ is considered to be an ideal discharge product of non-protonic Li-O_2_ batteries (Fig. 4a and b).^41^

In order to verify the reaction products, XRD, XPS, and SEM tests were performed. The XRD results showed that after the discharge, the characteristic peak of Li_2_O_2_ uniformly distributed over carbon sheets, indicating that the discharge product of this reaction process was reversible Li_2_O_2_, and as the charging process progressed, Li_2_O_2_ was decomposed. After the charging was completed, the characteristic peak of Li_2_O_2_ on the surface of the carbon sheet disappeared, indicating that Li_2_O_2_ had been completely decomposed (Fig. 4c). The XPS spectrum of Li 1s of the carbon sheet showed that there were two characteristic diffraction peaks, the peak at 55.3 eV was Li_2_O_2_, and the peak at 56.2 eV was Li_2_CO_3_, indicating that the main reaction product of the entire discharge reaction process was Li_2_O_2_, and it was also accompanied by certain side reactions, generating Li_2_CO_3_. As the charging process progressed, Li_2_O_2_ was decomposed, the characteristic peak disappeared, and only the undecomposed Li_2_CO_3_ remained (Fig. 4d). SEM images reveal that the pristine carbon sheet possesses a relatively smooth surface (Fig. 4e). After discharge, abundant crescent-shaped discharge products formed on the carbon sheet surface (Fig. 4f), combined with the literature and XRD test results, it was proved that it was Li_2_O_2_. After charging, these discharge products decomposed, and the carbon sheet surface was restored to a relatively smooth morphology (Fig. 4g), indicating that the Li-O_2_ battery assembled with Ni/Co_2_N@NCNTs as the positive electrode catalyst has good reversibility.

## Conclusions

In summary, by selecting MOF templates with high specific surface area and high porosity and preparing metal-coated nitrogen-doped carbon nanotube materials through self-catalytic carbon nanotubes, a large number of exposed active sites can be obtained, the conductivity of the material can be improved, and the electron migration rate can be accelerated. Under the conditions of current density of 100 mA g^−1^ and limit specific capacity of 1000 mAh g^−1^, the overpotential of Ni/Co_2_N@NCNTs battery is 0.76 V, while the overpotential of Ni@NCNTs battery is 1.32 V and the overpotential of Co_2_N@NCNTs battery is 1.18 V. This strategy provides a direction for the preparation of highly active bifunctional catalysts.

## Author contributions

Yajun Zhao: methodology, writing – original draft, conceptualization, writing – review & editing. Cen Wang: validation, investigation. Zhipeng Yao: validation, investigation. Guocai Zheng: validation, investigation. Guofei Li: validation, investigation. Xiaojing Li: validation, investigation.

## Conflicts of interest

The authors declare no competing financial interests.

## Supplementary Material

RA-OLF-D6RA06535J-s001

## Data Availability

All data supporting the findings of this study are included in the published article and its supplementary information (SI). Supplementary information is available. See DOI: https://doi.org/10.1039/d6ra06535j.
