# Supplementary material for: Self-growth of nitrogen-doped carbon nanotube composites using MOFs as templates and their application in Li-O2 batteries
Source: RSC Adv. 2026 Aug 26;16(42):50664–70. doi: 10.1039/d6ra06535j (PMC13508250; doi:10.1039/d6ra06535j)
Supplement: Supplementary file 1 [file RA-016-D6RA06535J-s001.pdf]

Supplementary Information (SI) for RSC Advances.
This journal is © The Royal Society of Chemistry 2026

Self-growth of nitrogen-doped carbon nanotube composites using MOFs as templates and their application in Li-O_2 batteries

Yajun Zhao[a,*], Cen Wang[a], Zhipeng Yao[a], Guocai Zheng[a], Guofei Li[a], Xiaojing Li[a]

[a] School of Automotive Applications and Rail Transit, Hefei Technology College, Hefei 230012, Anhui, China.

* Corresponding authors: zyj@htc.edu.cn

Experimental Section:

(1) Preparation of Co-BTC

0.392 g of cobalt acetate was dissolved in a mixture of 20 mL of deionized water and 20 mL of ethanol. 2.4 g of polyvinyl pyrrolidone was added under stirring. 0.72 g of 1,3,5-benzenetricarboxylic acid was dissolved in a mixture of 20 mL of deionized water and 20 mL of ethanol. The 1,3,5-benzenetricarboxylic acid solution was added dropwise to the Co salt solution under stirring. The mixture was stirred for 2 h, aged at room temperature for 24 h, centrifuged, washed, and dried.

(2) Preparation of Co_2N@NCNTs

Take a certain amount of Co-BTC and a certain amount of dicyandiamide according to the mass ratio of Co-BTC to dicyandiamide of 1:20, and place the two in two quartz boats respectively. Dicyandiamide is placed in the upwind of the tube furnace, and the product is placed in the downwind. Calcination is carried out in an Ar gas atmosphere, firstly heating to 400°C at 10°C/min, keeping the temperature for 2h, and then heating to 800°C at 10°C/min, keeping the temperature for 4h, and then obtaining the Co_2N@NCNTs composite material.

(3) Preparation of Ni@NCNTs

0.485 g of $NiCl_2$•$6H_2O$ was dissolved in 40 mL of deionized water and 40 mL of ethanol. 0.2315 g of ammonium fluoride and 0.546 g of urea were added under stirring. The mixture was stirred for 30 min and transferred to a 100 mL Teflon reactor. The mixture was hydrothermally reacted at 180 °C for 12 h. After cooling to room temperature, the mixture was centrifuged, washed, and the precipitate was collected and dried. A certain amount of dicyandiamide was weighed according to the mass ratio of product to dicyandiamide of 1:20. The two were placed in two quartz boats respectively.

The dicyandiamide was placed in the upwind side of the tube furnace and the product was placed in the downwind side. The mixture was calcined in an Ar atmosphere. The temperature was first increased to 400 °C at 10 °C/min and kept at this temperature for 2 h. Then, the temperature was increased to 800 °C at 10 °C/min and kept at this temperature for 4 h. The Ni@NCNTs composite material was obtained.

Material Characterization

The D/MAX2500V X-ray diffractometer (XRD) is selected to test the phase of the prepared material, the scanning speed is 10º/min, and the scanning range is 20-90º. The Gemini 500 field emission scanning electron microscope (SEM, Carl Zeiss Jena) is used to study the microscopic morphology of the sample. The internal structure and element distribution of the material is characterized by a transmission electron microscope (TEM, JEM-2100F, Hitachi), combined with selected area electron diffraction (SAED) and energy dispersive X-ray (EDX) spectroscopy tools. The ESCALAB250 X-ray photoelectron spectrometer (XPS) is used to analyze the surface composition of the sample and the valence of each element. The specific surface area of the material is calculated through the N_2 adsorption and desorption test of the material at 77 K.

Battery performance test

Use CT-3008 Neware battery test system to test the battery performance of materials. The battery uses lithium sheet as the negative electrode, glass fiber filter membrane (Whatman grade GF/D) as the diaphragm, 1 mol/L LiTFSI solution (with TEGDME as the solvent) as the electrolyte, and the catalyst-loaded carbon paper as the oxygen electrode. Assemble 2032-type button batteries in an argon glove box. The oxygen electrode was prepared by KB, catalysts, and PVDF with a weight ratio of 6: 3: 1. The load of catalyst and KB on the carbon paper was about 0.5±0.1 mg cm^{-2}.The catalyst loading amount on each cathode was accurately measured before electrochemical testing. The specific discharge capacity and applied current density were calculated based on the actual mass of the catalyst (Ni@NCNTs, Co_2N@NCNTs, or Ni/Co_2N@NCNTs) loaded on the electrode.

Battery charge and discharge performance test: current density is 100 mA g^{-1}; voltage range is 2.0-4.5 V (vs. Li^+/Li). Battery cycle performance test: The current density is 500 mA g^{-1}; the voltage range is 2-4.5 V (vs. Li^+/Li); the specific charge-discharge capacity is limited to 1000 mA h g^{-1}.

Cyclic voltammetry (CV) test and electrochemical impedance spectroscopy (EIS) test were performed on assembled button cells using an Autolab instrument. The scan rate of the CV test was 0.1 mV/s, and the voltage range was 2-4.5 V (vs. Li+/Li). The impedance scanning frequency range is 100 kHz-0.01 Hz, and the amplitude is 10 mV.

The battery charging and discharging process mechanism analysis uses XRD and SEM to analyze the surface phase and morphology changes of the carbon sheet after charging and discharging.

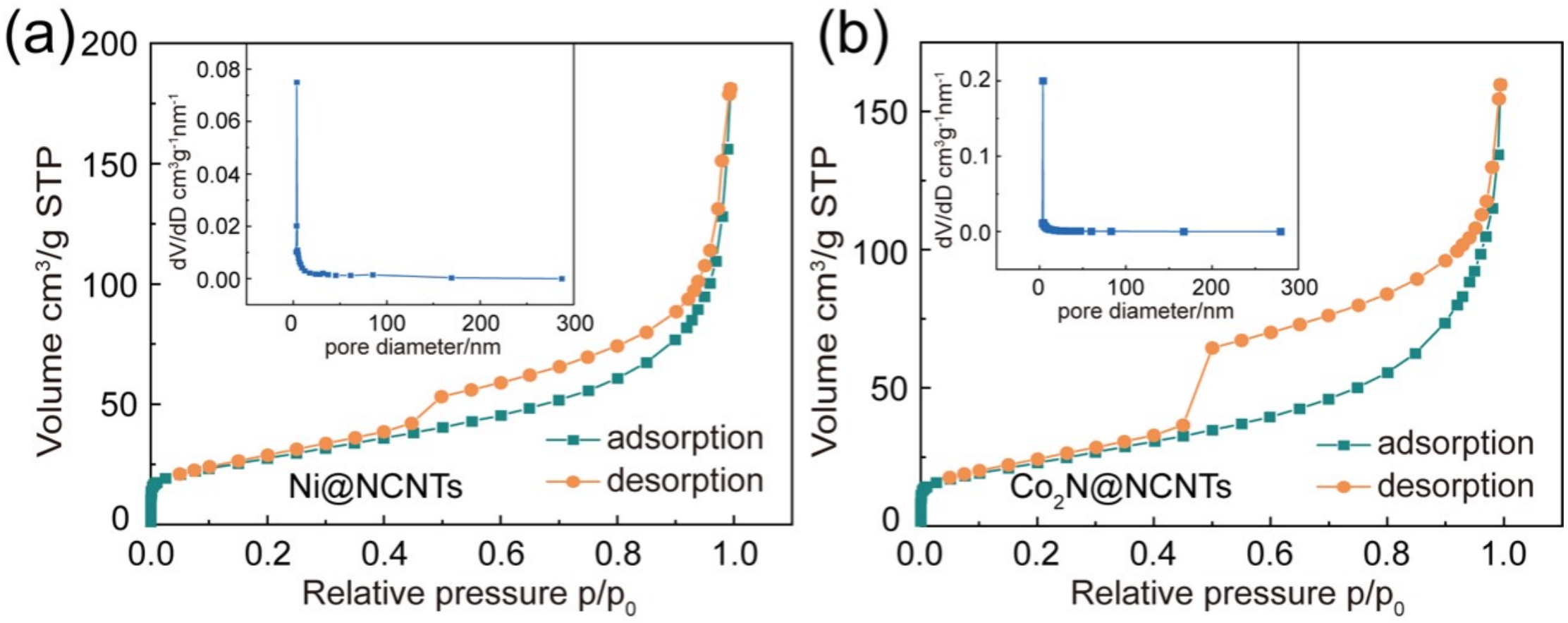

Fig. S1 N_2 adsorption-desorption curves of: (a) Ni@NCNTs; (b) Co_2N@NCNTs.

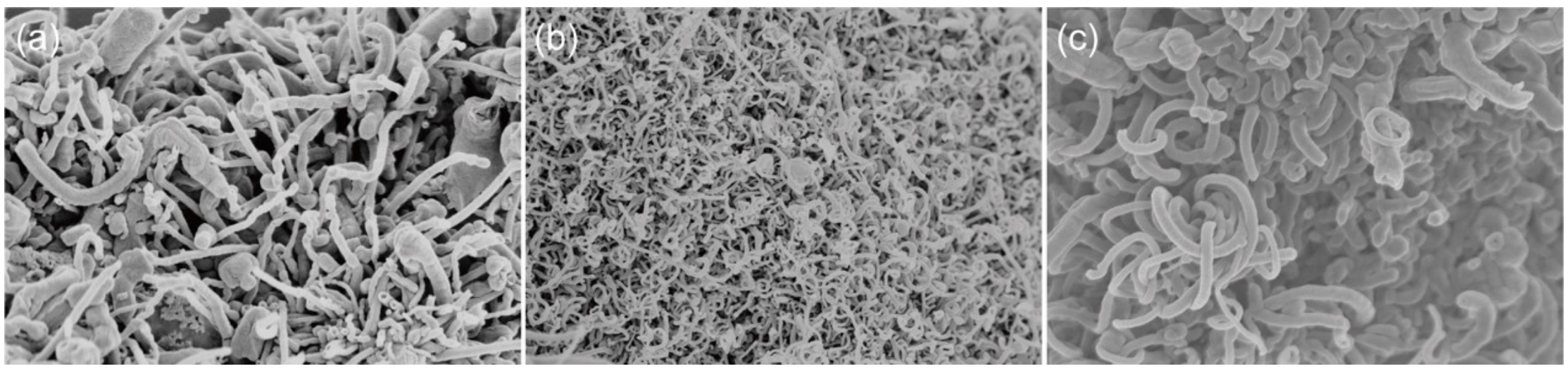

Fig. S2 SEM images of: (a) Ni@NCNTs; (b) Co_2N@NCNTs; (c) Ni/Co_2N@NCNTs.

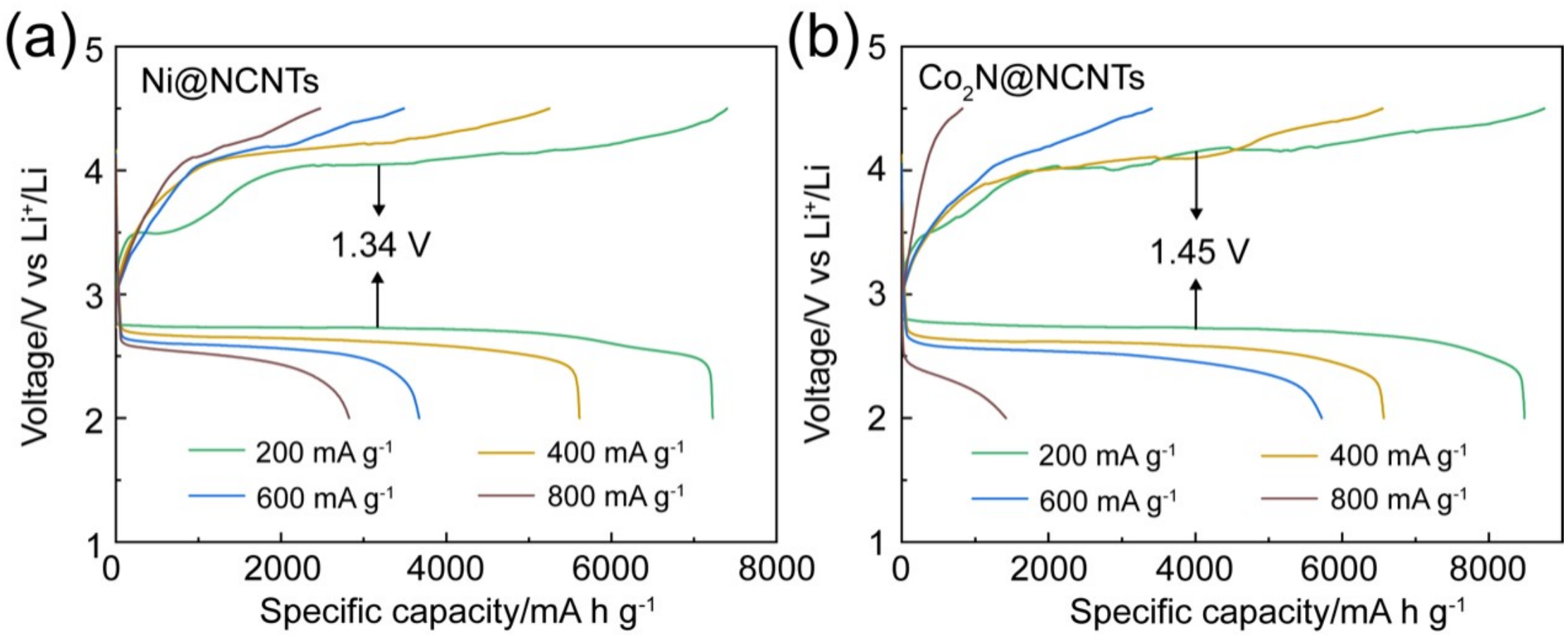

Fig. S3 Rate performance diagram of: (a) Ni@NCNTs; (b) Co_2N@NCNTs

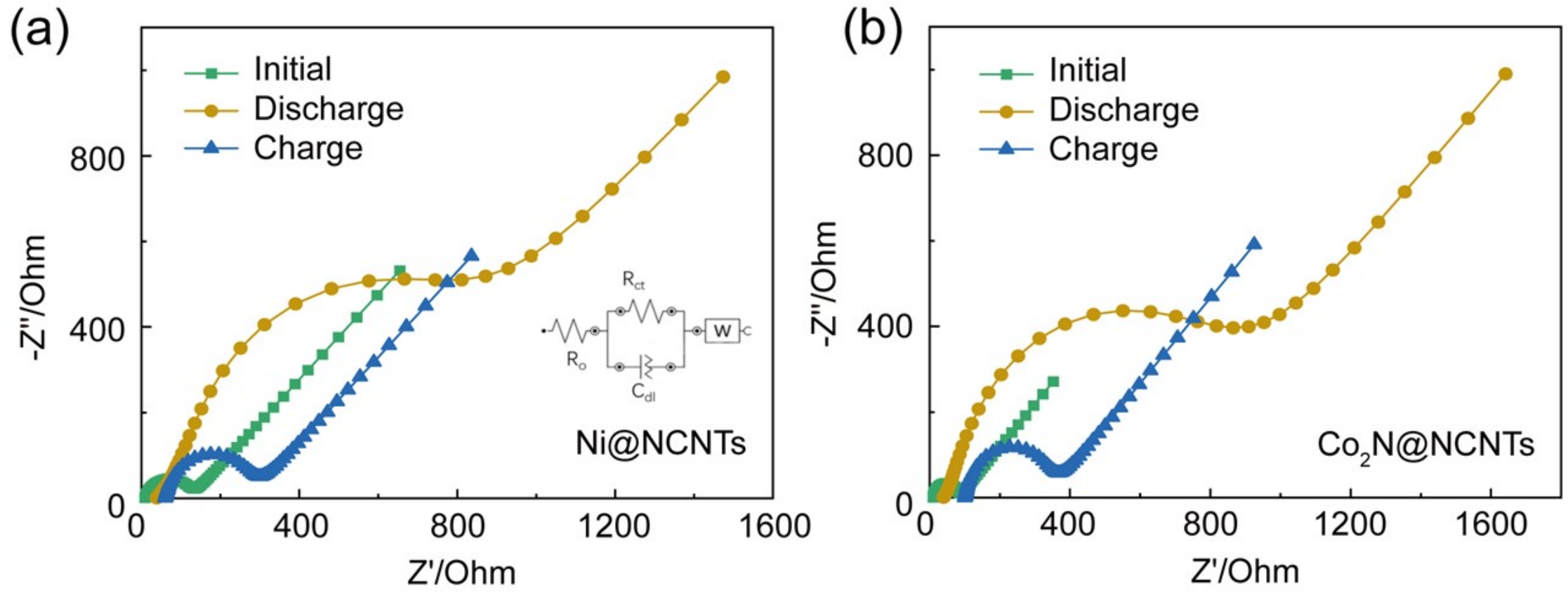

Fig. S4 The impedance change diagram of the two materials in different states (including the initial state, fully discharged state , and fully charged state) : (a) Ni@NCNTs; (b) Co_2N@NCNTs

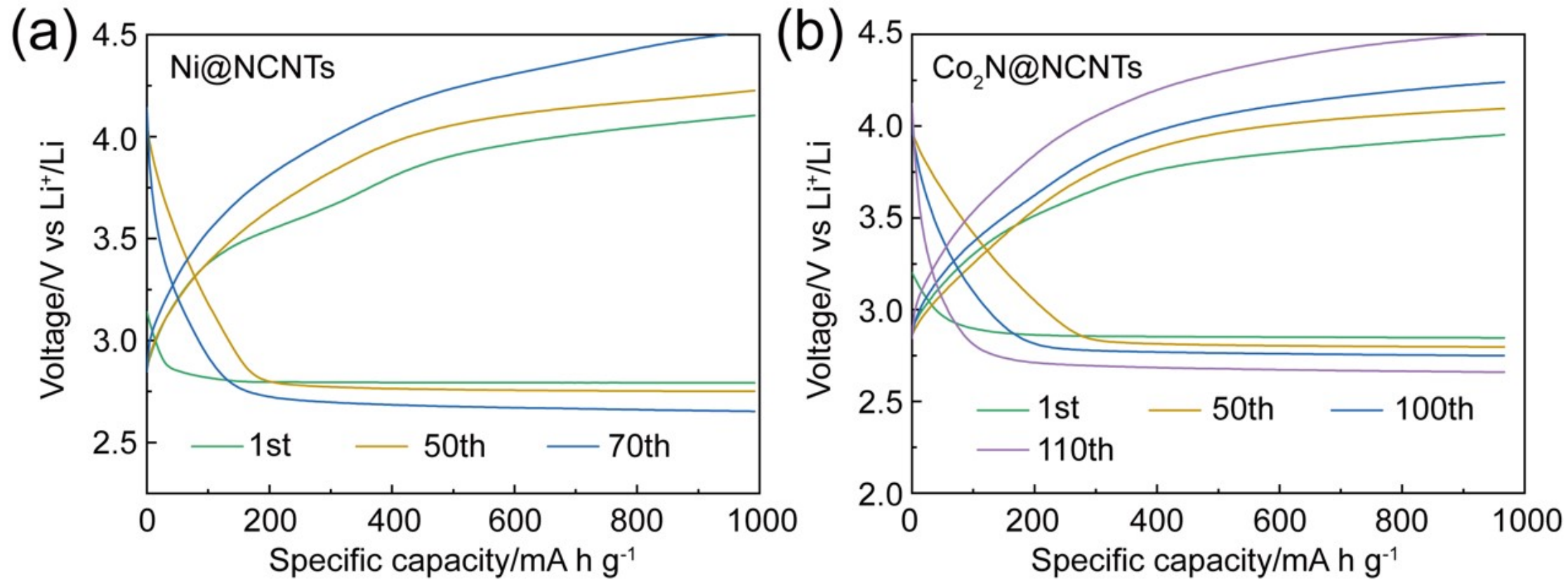

Fig. S5 The battery cycle diagram of the two materials: (a) Ni@NCNTs; (b) Co_2N@NCNTs
